# A Retrospective Analysis of Emergency Versus Elective Surgical Outcomes in Colon Cancer Patients: A Single-Center Study

**DOI:** 10.3390/jcm13216533

**Published:** 2024-10-30

**Authors:** Roxana Loriana Negruț, Adrian Coțe, Adrian Marius Maghiar

**Affiliations:** 1 Department of Medicine, Doctoral School of Biomedical Sciences, Faculty of Medicine and Pharmacy, University of Oradea, 410087 Oradea, Romania; popa.roxanaloriana@student.uoradea.ro (R.L.N.);; 2 County Clinical Emergency Hospital Bihor, 410087 Oradea, Romania; 3 Department of Surgical Disciplines, Faculty of Medicine and Pharmacy, University of Oradea, 410073 Oradea, Romania

**Keywords:** colon cancer, emergency surgery, colon cancer complications, inflammatory markers

## Abstract

**Introduction:** Emergency surgical interventions for colon cancer are often associated with poorer outcomes compared to elective surgeries due to the advanced state of the disease and the urgency of intervention. This retrospective study aimed to evaluate the management of emergency management of colon cancer and to evaluate differences in patient outcomes. Conducted at a single surgical emergency center, the study analyzed 182 cases, focusing on demographics, tumor characteristics, surgical methods and patient outcomes. **Material and Methods:** A retrospective observational study was conducted, involving 182 cases who underwent surgery for colon cancer in a single surgical emergency center. Data was collected from hospital records, encompassing demographic details, tumor characteristics, surgical intervention detail and outcomes, alongside with inflammatory profiles. Statistical analyses included descriptive statistics and *t*-tests with standard significance at *p* < 0.05. **Results:** The study showed that emergency cases had significantly poorer in-hospital survival rates (75.42%) compared to elective surgeries. Inflammatory markers such as Neutrophil-Lymphocyte Ratio, Platelet-Lymphocyte Ratio were higher in emergency cases, suggesting heightened systemic stress. Emergency surgery was also associated with a higher incidence of ostomy and postoperative complications. **Conclusions:** Emergency surgery for colon cancer is linked to more advanced tumors, increased physiological stress and lesser clinical outcomes. Early detection strategies and active targeted screening could reduce the need for emergency interventions. Future research should focus on early diagnosis protocols and enhancing public health strategies to minimize emergency presentations, thereby leading to better outcomes for colon cancer patients.

## 1. Introduction

Colon cancer is a public health concern, according to World Health Organization and Global Center Observatory, ranking 4th in prevalence worldwide, and 5th as cause of death among cancers [1]. Emergencies in colon cancer involve urgent surgical intervention due to life-threatening complications, such as bowel obstruction, perforation and acute bleeding [2]. These critical situations raise concerns about the need for immediate decision-making, which poses significant challenges due to the complexity of the disease. These situations associate a high risk of morbidity and mortality. A better understanding of the outcomes of emergency colon surgery can improve patient prognosis and assist surgeons in making immediate decisions. Existing medical literature indicates that patients undergoing one-stage emergency surgery have lower survival rates compared to those treated electively, often due to late-stage diagnosis and the severity of complications at presentation [3,4]. 

A recent meta-analysis of 28 studies, involving 353,686 participants demonstrated that emergency surgery had higher hospital mortality and higher risk of postoperative complications compared to elective surgery [5]. However, the number of lymph node harvested was comparable between emergency and elective procedures [5].

Recent research suggests that awareness level about colon cancer is still suboptimal, indicating the need for intensified efforts and targeted strategies to improve screening practices [6,7,8]. In Romania, the lack of a national screening program for colon cancer leads to late patient presentations, similar to other countries without such programs [9,10]. Currently, Romania does not have a fully implemented national screening program, though there are pilot projects underway. 

Regarding the demographic data, a recent systematic review and meta-analysis evaluating colorectal cancer showed that 17 studies reported disparities in colorectal screening between rural and urban populations, 12 studies highlighted treatment inequities, and 8 studies focused on differences in staging [11].

The study’s objective is to evaluate and compare in-hospital survival rates, complication rates and length of hospital stay, assess inflammatory profiles in both groups, procedural differences between emergency and elective surgical cases for colon cancer. Also, we aim to analyze the demographic data of patients, tumor characteristics including tumor stage and location, that contribute to emergency presentations. By focusing on these aspects, we aim to identify critical factors that contribute to poor outcomes in emergency cases and explore avenues for improving early detection and management to prevent the need for emergency interventions.

## 2. Materials and Methods

A retrospective observational study was conducted, at a single surgical center from Clinical Emergency Hospital Bihor. It involved patients who underwent surgery for colon cancer between January 2019 and December 2022. Data were collected from hospital records, encompassing demographic details, tumor characteristics, surgical approaches and postoperative outcomes.

Data collected included demographics (age, gender, geographical background), emergency diagnostics, tumor characteristics (location, stage, grading, histopathologic type), surgical interventions and weather an ostomy or anastomosis was performed, inflammatory markers (Neutrophil-lymphocyte Ratio, Platelet-lymphocyte Ratio, White Blood Cell counts) and outcomes (in-hospital survival, length of hospital stay and surgical complications).

### 2.1. Study Population

Patients who were initially diagnosed with colon cancer and underwent surgery at a single Surgery Department of Clinical Emergency Hospital Bihor that presented between January 2019 and December 2022 were included in the study. The hospital, which primarily serves as an emergency care facility, is also equipped to handle elective surgeries, due to the hospital’s status as the main referral center. Consequently, the study includes both emergency and elective surgical cases. We excluded patients that were previously operated for colon cancer and also those that underwent palliative surgery without resection. Patients who underwent either emergency or elective surgery for colon cancer were included in the study. Patients requiring emergency surgery had acute complications of colon cancer such as bowel obstruction or perforation, while patients who underwent elective surgery were those diagnosed and scheduled for surgery following routine diagnosis and staging.

### 2.2. Sampling Process

The sampling process was consecutive, meaning all eligible patients presenting during the study period who met the inclusion and exclusion criteria were included. This ensured a continuous and unbiased selection of cases for analysis.

### 2.3. Data Collection

Data were collected from hospital records, encompassing demographic details, tumor characteristics, surgical approaches and postoperative outcomes.

Data collected included demographics (age, gender, geographical background), emergency diagnostics, tumor characteristics (location, stage, grading, histopathologic type), surgical interventions and weather an ostomy or anastomosis was performed, inflammatory markers (Neutrophil-lymphocyte Ratio, Platelet-lymphocyte Ratio, White Blood Cell counts) and outcomes (in-hospital survival, length of hospital stay and surgical complications). The inflammatory markers were measured before surgery, up to 3 days prior the intervention.

### 2.4. Statistical Analysis

The statistical methods included descriptive analysis, *t*-test performed for continuous data, Chi-Squared tests for categorical variables and contingency tables, using Microsoft Excel for Mac version 16.90.2 and JASP 0.19.1 (Apple Silicon) software [12]. 

Analysis of Covariance (ANCOVA) was employed to compare inflammatory markers such as NLR between emergency and elective surgery groups, being able to control for the effects of age, sex, and tumor characteristics, ensuring that differences in inflammatory markers between surgery groups are not confounded by these factors.

Logistic regression analysis was used to assess the association between emergency surgery and in-hospital mortality, adjusting for confounders such as age, sex, and tumor staging. Odds ratios (OR) and 95% confidence intervals (CI) were calculated to interpret the strength of these associations. Model performance was assessed using Nagelkerke R^2^, McFadden R^2^, and the Area Under the Curve (AUC) for the receiver operating characteristic (ROC) curve. 

Statistical significance was set at *p* < 0.05. The results are presented as counts and percentages for categorical data, mean values and standard deviations (SD) for normally distributed variables.

Emergency surgery is an operation performed in an urgent setting, due to acute complications of colon cancer, while elective surgery is performed after diagnostic, staging and treatment planning. The emergency surgery indications are bowel obstruction, acute peritonitis, perforation and uncontrolled bleeding from the tumor. 

## 3. Results

A total of 182 patients who underwent surgery for colon cancer at our clinic during the 4-year timeframe. Among them, 118 underwent emergency surgery due to undiagnosed colon cancer complications. 

The average age was comparable between the emergency (Mean: 69.66 years) and the elective group (70.47). There is a slight male predominance (59.34%), and a higher proportion of emergency surgeries for patients that originated from rural areas, indicating potential barriers to timely diagnosis in these populations. The demographic data is presented in Table 1.

The leading cause for emergency surgery was bowel obstruction (81 out of 118 cases) fallowed by fecaloid peritonitis due to tumoral or diastatic perforation (21 cases), as seen in Table 2. This aligns with the urgency required to relieve a life-threatening condition such as obstruction explains the high rate of stoma procedures in this situations.

**Figure 1:**
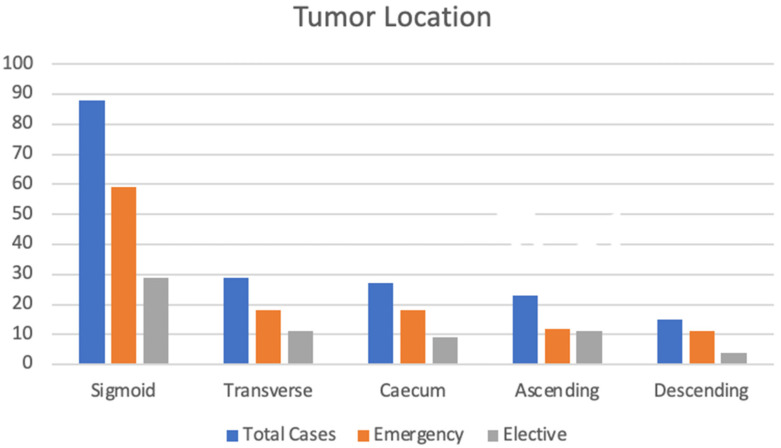
Distribution of tumor location by total cases, emergency surgery and elective surgery.

The location of the tumor in emergency cases was predominant with the sigmoid colon (59 cases), followed by transverse colon (18 cases) and caecum (18 cases). The same location pattern was encountered for elective surgery, shown in the bar chart Figure 1. 

The tumors in emergency cases were in advanced stages (stage III and IV), representing 49% cases. This indicates that tumors identified in emergencies are often more advanced, which could contribute to the urgency and higher associated mortality. The stages of the encountered tumors in each situation are presented in Table 3. 

Both preoperative Neutrophil-Lymphocyte Ratio (NLR) and Platelet-Lymphocyte Ratio (PLR) are significantly elevated in emergency cases, with *p*-values indicating a clear difference (NLR: *p* = 0.013, PLR: *p* = 0.0028). Elevated NLR and PLR are often markers of physiological stress, systemic inflammation, or severe infection, further supporting their association with the need for emergency interventions. The Cop-NLR score is significantly different between emergency and non-emergency cases, suggests that emergency cases have different inflammatory profiles, offering a marker for surgical urgency. The CoP-NLR is the combination of platelet count and NLR. The COP-NLR score was determined using data collected on the day of admission, with patients assigned a score of 2 if both platelet count (>30 × 10^4^/mm^3^) and NLR (>3) were elevated, a score of 1 if only one of these values was elevated, and a score of 0 if both were below the set limits. The cut off values were taken from preexisting studies [13]. The higher the NLR and PLR are usually indicator of poor prognosis and systemic inflamation due to acute complications. This suggests a heightened inflammatory response in emergencies. The *p*-value of 0.012 from the Chi-sqared test suggests that there is a statistically significant diference in the distribution of CoP-NLR scores between the emergency and elective cases. The distribution shows a higher concentration of CoP-NLR scores of 2 in emergency cases, suggesting that patients in emergency situations tend to have higher inflammatory response, becoming a meaningful indicator of patient status, showing a more critical profile of the patient. The normal range values for Neutrophils are 1.8–7.5 × 10^3^/μL, for WBC is 4.0–10.0 × 10^3^/μL and PLT 150–450 × 10^3^/μL. Table 4 presents a comparison of inflammatory markers between emergency and elective cases, demonstrating significant differences.

As seen in Table 5, ANCOVA was conducted to compare the effect of surgery type (emergency vs. elective) on NLR while controlling for age, sex, and tumor characteristics. There was a significant effect of surgery type on NLR, F(1, X) = 5.000, *p* = 0.027. However, the covariates age (*p* = 0.980), sex (*p* = 0.217), tumoral infiltrated ganglions (*p* = 0.727), and staging (*p* = 0.553) did not show statistically significant effects on NLR. Similar results were found for PLR.

This figure presents the adjusted mean NLR values between emergency and elective surgeries, controlling for covariates such as age, sex, tumoral infiltrated ganglions, and tumor staging. The analysis shows a significant difference in NLR between the two surgery types after accounting for these factors.

The white blood cell count (WBC) is significantly higher in emergency cases, with a mean of 13.23/10^3^/µL vs. 9.89/10^3^ µL for non-emergencies. This may be indicative of infection or severe inflammation, which are common contributors to the need for emergency surgery. The *p*-value for WBC differences between emergency and non-emergency cases (0.004) indicates a significant distinction, suggesting that elevated WBC can be a predictor of the need for urgent surgery. Table 6 illustrates the significant difference between the WBC counts.

The choice of surgical intervention, presented in Table 7, was influenced by the site of the primary tumor. Partial colectomy was performed in 54.2% of cases and right colectomy in 28%, being the most common surgeries performed. Ostomies were performed in 78 emergency cases, compared to 12 from elective cases, indicating a tendency for ostomy during urgent scenarios. All surgical procedures were performed under open approach.

Ostomy procedures are much more common in emergency situations (78 cases vs. 12 non-emergency), indicating a preference for more immediate, simpler methods to secure bowel function in critical situations. Anastomosis is preferred in non-emergency situations, likely because of a more favorable clinical environment that allows safe healing of the bowel, to reduce the anastomotic fistula rates. Table 8 shows the distribution of bowel integrity outcomes after surgery, with a significantly higher frequency of anastomosis. In Table 9, the type of anastomosis performed are compared between emergency and elective cases.

Table 10 provides a comparison of the mean number of nodes harvested and tumoral infiltrated nodes between emergency and elective cases. There are no significant differences in the average number of ganglions or infiltrated ganglions between emergency and non-emergency cases. This indicates that while the tumor burden may differ, the extent of lymph node involvement is comparable. For colon cancer resection, the generally accepted standard, recommended by major guidelines is to examine at least 12 lymph nodes. We must acknowledge that 113 cases out of 182 had under this threshold, fewer than 12 nodes being retrieved.

The most common histopathological type is NOS adenocarcinoma (145 cases), followed by mucinous adenocarcinoma (32 cases), as presented in Table 11.

The G2 grading is the most common across both emergency and elective cases, but with a higher prevalence in emergency surgeries. This suggests that tumors with moderate differentiation are often detected at an urgent stage, possibly due to their tendency to present more aggressive or symptomatic characteristics compared to lower-grade tumors. The chi-squared test reveals no significant difference in grading between emergency and non-emergency surgeries (*p*-value = 0.80). The results are presented in Table 12.

The average tumor size, as presented in Table 13, is slightly larger in elective cases (5.61 cm vs. 5.43 cm), but the range and variability are higher for emergency patients, suggesting that emergency cases may present with more diverse and possibly later-stage tumors.

The data indicates that a large portion of emergency cases (49) proceed to surgery within a day, compared to 19 cases in elective cases. This stresses the importance of rapid response capabilities in emergency surgical care, as delays can directly impact patient outcomes. Hospitals with high volumes of such cases need efficient triage and operating room readiness. Table 14 displays the timing of surgery after admission.

As shown in Table 15, the mean length of stay is slightly longer for emergency cases (12.34 days vs. 11.95 days for non-emergencies). The greater variability in emergency length of stay (standard deviation 10.26) suggests that post-operative recovery is less predictable and more complex for emergency surgeries, possibly due to the unstable condition of patients on admission. 

The postoperative outcomes, presented in Table 16, indicated that survival rates during hospital stay differed significantly between emergency and elective surgery. Elective cases had a survival rate of 93.75%, while emergency cases demonstrate a notably lower survival rate of 75.42%. This indicates that emergency surgery is associated with a notably higher risk of mortality during the hospital stay. The *p*-value was calculated using t test (0.004) for the difference in mortality between emergency and non-emergency cases confirms that this difference is statistically significant. This emphasizes the critical importance of rapid and effective interventions in emergency situations.

We performed logistic regression to assess the association between emergency surgery and patient outcomes (death during hospital stay). The model included Emergency status (binary: Yes/No), age, sex, and tumor staging as predictor variables. To account for potential confounders, we adjusted for age, sex, and tumor staging. The model’s goodness-of-fit was assessed using Nagelkerke R^2^, and odds ratios (OR) with 95% confidence intervals were calculated to interpret the effects of each variable. The model performance was evaluated using Area Under the Curve (AUC), sensitivity, and specificity.

The logistic regression model revealed that emergency surgery was a significant predictor of in-hospital death, with an odds ratio of 5.272 (95% CI: 0.544–2.781, *p* = 0.004), indicating that patients who underwent emergency surgery were over five times more likely to die during hospitalization compared to those who underwent elective surgery, as presented in Table 17 and Table 18. 

This tables show the confusion matrix for predicted vs. observed outcomes and the performance metrics (AUC, sensitivity, specificity, precision) of the logistic regression model predicting in-hospital mortality. See Table 19.

As seen in Table 20, age was also a significant factor, with an odds ratio of 1.049 (95% CI: 0.006–0.090, *p* = 0.025), suggesting that the odds of in-hospital death increased slightly with age. However, sex and tumor staging did not significantly affect mortality, with *p*-values of 0.594 and 0.180, respectively.

This figure presents the summary of the logistic regression model, including goodness-of-fit statistics (AIC, BIC, McFadden R^2^, Nagelkerke R^2^) and coefficients for the predictors (Emergency binary, age, sex binary, and staging). Odds ratios, Wald tests, and confidence intervals are provided to interpret the significance of each variable.

The overall model fit was modest, with a Nagelkerke R^2^ of 0.155, indicating that approximately 15.5% of the variance in death during hospitalization could be explained by the model. The model’s discrimination ability was fair, with an AUC of 0.737, while the specificity was high at 0.987, but the model demonstrated low sensitivity (0.030) for predicting death.

Complication rates are presented in Table 21. Complications were also higher for emergency surgery, 30% of patients experiencing surgical complications as evisceration, anastomotic fistula or diastatic fistula.

In Table 22, the mean duration of postoperative complications is presented based on surgery type. Complications tend to occur later in patients who underwent left colectomy (average 9.71 days), compared to right hemicolectomy and segmental resection. This could indicate specific post-surgical risks associated with left-sided procedures, potentially requiring longer monitoring post-surgery.

## 4. Discussion

The study aimed to explore the differences in outcomes between emergency and elective cases of colon cancer by analyzing data records from a single center on a period of 4 years. The findings demonstrated key distinctions in patient characteristics, inflammatory profiles, surgical methods and postoperative outcomes between the two groups.

The findings of the study indicate that patients that undergo emergency surgery for colon cancer present with advanced disease stages, increased inflammatory markers and worse clinical outcomes. The survival rate for patients undergoing emergency surgery was significantly lower (75.42%) compared to those who underwent elective surgery. This disparity outlines the increased risk in emergency situations, where patients present themselves at the hospital with severe complications like bowel obstruction or peritonitis, fallowing the need for immediate surgical intervention. 

The higher number of cases from rural areas suggest the potential barriers that patients encounter for cancer screening and diagnosis. Strategies to enhance healthcare access in rural regions could reduce the number of emergency presentations.

The findings of this study are consistent with the existing literature, suggesting that emergency interventions occur when patients have progressed to critical conditions, with higher morbidity and mortality. 

The sigmoid colon was most frequently associated with emergency presentations, due to anatomical and functional factors influencing the risk of obstruction. The high prevalence of advanced stages in emergency setting highlights the need for improved early detection, mostly for populations at risk.

The high frequency of stoma in emergency cases reflects a pragmatic approach to stabilizing patients in critical conditions, prioritizing life-saving procedures. Anastomosis is preferred in elective settings for the benefits of intestinal restauration, but when performed under emergency conditions for complications of the pathology, the risk for complications is elevated. Clinical situations involving perforation can be particularly severe, especially when free fecal peritonitis occurs, becoming crucial to emphasize the importance of balancing life-saving surgical interventions with adherence to oncological principles [14]. Even though there is evidence that supports that presence of fecal matter in the large intestine does not affect the rate or severity of anastomotic dehiscence [14,15,16,17] and numerous prospective and retrospective studies on anastomosis in obstructive left colon cancer reported similar anastomotic leak incidence with the elective surgeries (2.2% to 12% for obstructive disease and 2–8% rate for elective surgeries) [18,19,20,21,22,23,24], the potential disastrous consequences of anastomotic leakage in vulnerable patients must be taken in consideration. Multiple factors related to patients’ condition and surgeon’s expertise must be carefully weighted before opting for colo-colonic anastomosis in emergency settings [14,21,22,25]. Resection with colostomy may be more suitable for patients considered at high risk or managed in emergency situations by less specialized surgeons [6].

The research available on obstructive right colon cancer is less comprehensive compared to that on the left colon, probably due to advantageous anatomical features, making right colectomy with primary anastomosis the standard approach. Several anatomical reasons contribute to this: hepatic flexure is simpler to mobilize and the flexibility of the small intestine, optimal blood supply, unlike certain vulnerable areas of the left colon [14,26].

The significantly higher levels of NLR and PLR and WBC in emergency cases emphasize the severity of these situations, suggesting systemic inflammations and physiological stress, this being linked to poorer surgical outcomes. The NLR and PLA are easily accessible biomarkers that can be effortlessly calculated in ward setting, without the need for changes in current practice. Current available literature indicates that preoperative NLR is strongly linked to poorer outcomes in patients undergoing emergency colon cancer surgery, suggesting that it could be useful in predicting a patient’s prognosis after procedure [27,28]. 

One of the strengths of this study is the relatively large sample size, data recorded being comprehensive, including demographic information, tumor characteristics and inflammatory profiles. This dataset enabled a comparation between emergency and elective cases. However, the retrospective nature and the fact that the sample is limited to a single center, limit the generalizability of the result, having potential biases related to data completeness and accuracy. Also, no comparison was made with those who were unsuitable for resection. Further studies should eliminate these limitations by adopting a prospective, multi-center approach to validate these findings.

## 5. Conclusions

The study illustrated that emergency surgical intervention for colon cancer is associated with less favorable outcomes compared to elective surgery. Patients that present with acute complications of colon cancer typically exhibit more advanced stages of cancer and are subjected to higher risk of surgical complications and mortality. The increased rates of complications and longer hospital stay state the complexity and severity of emergency cases. The findings emphasize the importance of early detection and timely surgical intervention to improve patient outcomes. 

Firstly, the prevalence of advanced-stage tumors in emergency settings underscores the need for enhanced early detection and screening strategies, particularly targeting high-risk populations, such as those in rural areas who may face barriers in accessing healthcare. Strengthening public health initiatives to facilitate routine screening and early diagnosis can significantly reduce emergency presentations.

Secondly, elevated inflammatory markers, such as NLR, PLR, WBC and CoP-NLR score were consistently linked with emergency cases, highlighting their potential role as predictive markers for clinical severity. Routine assessment of these markers in clinical practice could be instrumental in early identification of patients at higher risk for severe complications, enabling timely interventions that may improve outcomes.

Thirdly, the differences in surgical approaches between emergency and elective cases, such as the increased reliance on ostomies, suggest the necessity for immediate stabilization in emergencies. To improve outcomes, efforts should be made to better prepare facilities and surgical teams to manage emergency cases effectively, potentially through specialized training and resource allocation to ensure prompt access to high-quality surgical care.

Additionally, the higher complication rates and prolonged hospital stays associated with emergency interventions indicate a need for more effective perioperative and postoperative management protocols for these patients. Enhanced monitoring and postoperative support could reduce complication rates and aid in recovery, ultimately improving survival outcomes.

Future research should focus on improving the management of emergency colon cancer surgeries and exploring strategies to reduce the need for emergency interventions through enhanced public health measures and early detection initiatives.Also, given the strong association between elevated inflammatory markers and adverse surgical outcomes, future research should focus on developing a screening or prediction model to identify patients at high risk, by integrating preoperative immune markers, clinical condicions and demographic factors to predict outcomes like postoperative complications, length of stay, short and long term survival By addressing these factors, the overall prognosis for colon cancer patients requiring surgical intervention can be significantly improved.

## Figures and Tables

**Table 1:** Demographic data.

Demographics		Total (n = 182)	Emergency (n = 118)	Elective (n = 64)	X^2^/t-Value	*p*-Value
Mean age (years)		69.95	69.66	70.47	−0.508	0.62
Age SD		±10.22	±10.15	±10.41		
Male Patients	Sex	108	66	42	X^2^ − 3.278	0.07
Female Patients		74	52	22		
Area Urban		101	60	41	X^2^ − 1.037	0.792
Area Rural		81	58	23		

SD—standard deviation; *n* represents the number of patients in each group. X^2^—Chi-sqare value; t value—*t* test value. n is the total number of cases.

**Table 2:** Emergency diagnostics at presentation.

Emergency Diagnostic	Cases
Bowel Obstruction	81 (68.64%)
Fecaloid Peritonitis	21 (17.8%)
Purulent Peritonitis	16 (13.56%)

**Table 3:** Staging of colon cancer. Wald Test Logistic Regression.

Stage	Total Cases	Emergency Cases	Elective Cases	z-Value	*p*-Value
Stage I	23	11	12	0.208	0.835
Stage II	71	48	23	−2.901	0.004
Stage III	52	34	18	−2.182	0.029
Stage IV	35	24	11	−2.143	0.032
Stage 0 (In situ)	1	1	0	−0.017	0.987

**Table 4:** Inflammatory markers.

Metric NLR and PLR		Emergency	Elective	*p*-Value	t Value
Mean NLR		9.37	6.02	0.013	−2.496
Mean PLR		350.64	237.2	0.002	−3.088
CoP-NLR	Score 0	11	16	0.012	X^2^ = 8.866
	Score 1	45	24		
	Score 2	62	24		

NLR—Neutrophil to Lymphocyte Ratio; PLR—Platelet to Lymphocyte Ratio; CoP-NLR score-Combination of Platelet Count and Neutrophil to Lymphocyte Ratio.

**Table 5:** ANCOVA Results for NLR Between Emergency and Elective Surgeries.

Cases	Sum of Squares	Mean Square	F	*p*
Emergency	377.329	377.329	5.000	0.027
Age	0.048	0.048	6.316 × 10^−4^	0.980
Sex	116.017	116.017	1.537	0.217
Tumoral infiltrated ganglions	9.261	9.261	0.123	0.727
Staging (0/I/II/III/IV)	26.652	26.652	0.353	0.553

**Table 6:** White Blood Cell count.

WBC Count Metrics	Total	Emergency	Elective	*p*-Value	t Value
Mean	12.06	13.23	9.89	0.004	−2.92
Standard Deviation	7.5	8.3	5.15		
Range	1.91–53.17	1.91–53.17	3.66–34.37		

**Table 7:** Type of surgery performed.

Surgery	Total Cases	Emergency Surgery	Elective Surgery	X^2^	*p*-Value
Partial Colectomy	96	64	32	3.935	0.269
Right Colectomy	58	33	25		
Left Colectomy	25	18	7		
Multiple resections/subtotal colectomy	3	3	0		

**Table 8:** Bowel integrity after surgery.

Bowel Integrity	Total Cases	Emergency Cases	Elective Cases	X^2^	*p*-Value
Anastomosis	92	40	52	37.220	<0.001
Ostomy	90	78	12		

**Table 9:** Type of anastomosis.

Anastomosis Type	Emergency	Elective	*p*-Value
L-L	6	8	<0.001
L-T	1	0	X^2^
T-L	7	3	46.113
T-T	26	41	

**Table 10:** Nodes surgical harvested and infiltrated ganglions.

Nodes	Total	Emergency	Elective	t Value	*p* Value
Mean Nodes	10.64	10.12	11.61	−1.118	0.265
Standard Deviation	8.6	8.06	9.5		
**Tumoral infiltrated Nodes**	**Total**	**Emergency**	**Elective**	−0.068	0.94
Mean Infiltrated Ganglions	1.45	11.44	1.47		
Standard Deviation	2.64	2.67	2.6		

**Table 11:** Histopathological results.

Histopathologic Type	Subtype	Cases
ADK (Adenocarcinoma)	NOS	145
ADK (Adenocarcinoma)	MUCINOUS	32
ADK (Adenocarcinoma)	CRIBRIFORM	2
ADK (Adenocarcinoma)	MULTIFOCAL	1
CARCINOM	ANAPLAZIC	1
MIXT MINEN (Neuroendocrine)	NON Neuroendocrine	1

**Table 12:** Grading of colon cancer.

Grading	Total Cases	Emergency	Elective	X^2^	*p*-Value
G1	8	5	3	5.621	0.585
G2	115	77	38		
G3	26	17	9		
Not graded (mucinous adk/carcinoma)	33	19	14		

**Table 13:** Tumor size measured on specimen.

Tumor Size	Total	Emergency	Elective	t Value	*p* Value
Mean	5.49 cm	5.43 cm	5.61 cm	−0.491	0.624
SD	2.51	2.38	2.73		
Range	1.5–15.0	1.5–15.0	1.7–14.0		

**Table 14:** Days from admission to surgery.

Timing of Surgery After Admission	Total Cases	Emergency	Elective	t-Value	*p*-Value
≤1 day after admission	68	49	19	2.234	0.027
>1 day after admission	114	69	45		

**Table 15:** Length of stay.

Length of Stay	Total	Emergency	Elective	t Value	*p* Value
Mean Length of Stay	12.20 days	12.34 days	11.95 days	0.275	0.071
Standard Deviation	9.00 days	10.26 days	6.09 days		

**Table 16:** Survival rate during hospitalization.

Emergency Status	Survival Rate in Hospital (%)	Death Rate in Hospital (%)	X^2^	*p*
Emergency	75.42% (89)	24.58% (29)	9.388	0.002
Elective	93.75% (60)	6.25% (4)		

**Table 17:** Confusion Matrix of the Logistic Regression Model for In-Hospital Mortality.

Observed	Predicted NO	Predicted YES	% Correct
**NO**	147	2	98.658
**YES**	32	1	3.030
**Overall % Correct**			81.319

**Table 18:** Performance Metrics of the Logistic Regression Model for In-Hospital Mortality.

Metric	Value
**AUC**	0.737
**Sensitivity**	0.030
**Specificity**	0.987
**Precision**	0.333

AUC- Area Under The Curve.

**Table 19:** Logistic Regression Model Summary for In-Hospital Mortality.

Model	Deviance	AIC	BIC	df	ΔX^2^	*p*	McFadden R^2^	Nagelkerke R^2^	Tjur R^2^	Cox & Snell R^2^
**M_0_**	172.313	174.313	177.517	181			0.000	0.000	0.000	0.000
**M_1_**	154.171	164.171	180.191	177	18.142	0.001	0.105	0.155	0.095	0.095

**Table 20:** Logistic Regression Model Coefficients for In-Hospital Mortality.

Model	Estimate	Standard Error	Odds Ratio	z	Wald Statistic	*p*-Value	Lower Bound	Upper Bound
**M_0_**	−1.507	0.192	0.221	−7.835	61.391	<0.001	0.152	0.323
**M_1_**	−7.049	1.814	8.684 × 10^−4^	−3.886	15.105	<0.001	0.000	0.030
**Emergency**	1.662	0.571	5.272	2.914	8.849	0.004	1.723	16.131
**Age**	0.048	0.021	1.049	2.236	4.998	0.025	1.006	1.094
**Sex**	0.219	0.411	1.245	0.532	0.283	0.594	0.556	2.787
**Staging**	0.288	0.215	1.334	1.341	1.798	0.180	−0.133	2.032

**Table 21:** Surgical complications.

Complication Type	Emergency	Elective
Evisceration	8	2
Anastomotic Fistula	3	3
Diastatic Fistula	3	0
Hematoma	1	0
Colon Necrosis	1	0
Spleen Necrosis	1	0
Fournier Gangrene	1	0

**Table 22:** Postoperative complications depending on type of surgery.

Surgery Type	Mean Days of Complications	Standard Deviation	Number of Cases
Left Colectomy	9.71 days	5.09	7
Right Hemicolectomy	6.80 days	2.17	5
Segmental Resection	7.20 days	4.02	10

## Data Availability

The datasets during and/or analyzed during the current study are available from the first and corresponding author on reasonable request.
